# Application of the combined technique of novel transseptal puncture guidewire and steerable sheath in left atrial appendage occlusion: a preliminary feasibility case series

**DOI:** 10.3389/fcvm.2026.1908230

**Published:** 2026-09-03

**Authors:** Mei Liang, Wen Jia Wang, Peng Yu Wang, Qing Gu

**Affiliations:** 1 Department of Cardiology, The First People’s Hospital of Yuxi City, Yuxi, China; 2 Faculty of Engineering, Data Science at the University of Bristol, Bristol, United Kingdom

**Keywords:** case series, coaxiality, left atrial appendage occlusion, sheath migration, transseptal puncture, transseptal puncture guidewire

## Abstract

**Objective:**

Transseptal puncture (TSP) is a critical step in left atrial appendage occlusion (LAAO) but presents certain technical challenges. Conventional puncture needles, due to their inherent rigidity, often cause unpredictable needle tip “bouncing” or upward migration at the moment of septal puncture, resulting in a high or suboptimal puncture site, which subsequently compromises the coaxial alignment between the sheath and the left atrial appendage (LAA). This study aims to evaluate the safety and feasibility of the novel transseptal puncture guidewire Accusafe™ used in combination with a steerable sheath Navigo™.

**Methods:**

In this case series, 20 consecutive patients undergoing a one-stop procedure (LAAO combined with atrial fibrillation ablation) were enrolled. The first 10 patients underwent conventional transseptal puncture, while the latter 10 underwent transseptal puncture using Accusafe™. The primary endpoints included first-pass puncture success rate, incidence of post-puncture site migration, and coaxiality assessed by the angle between the delivery sheath and the central axis of the LAA on angiography.

**Results:**

Transseptal puncture was successfully performed in all patients. The application of Accusafe™ in combination with a steerable sheath significantly reduced sheath migration, achieving a first-pass puncture success rate of 100%. Furthermore, Accusafe™ significantly improved sheath-LAA coaxiality, effectively simplified the occluder delivery and deployment process, shortened procedure time, and ensured an ideal occlusion outcome.

**Conclusion:**

Transseptal puncture via the Accusafe™ guidewire with steerable sheath improves first-pass puncture success rate, enhances puncture site accuracy, and optimizes sheath–LAA central axis coaxiality, thereby improving procedural efficiency and occlusion efficacy. These findings support further validation of its clinical value in larger, randomized controlled studies.

## 1 Introduction

Transseptal puncture (TSP) is a critical step in percutaneous left atrial appendage occlusion (LAAO) (1, 2). Achieving an ideal puncture site within the fossa ovalis is essential to establish stable coaxial alignment between the delivery sheath and the long axis of the left atrial appendage (LAA), which directly influences device implantation success and the incidence of complications such as peri-device residual shunting (3, 4). However, the widely used technique employing fixed-curve sheaths combined with puncture needles presents inherent technical challenges in achieving optimal coaxiality during LAAO procedures. Due to the inherent rigidity of the puncture needle, an unavoidable ’spring-back’ motion or upward displacement of the needle tip frequently occurs as the operator advances the needle to penetrate the interatrial septum. This often causes the sheath assembly to deviate from the intended puncture site, resulting in a suboptimal trajectory (5, 6). Such deviations in puncture location typically leads to difficulties in device delivery or constrained deployment. This increases the procedural burden of intraoperative adjustments and significantly prolongs procedure time. In severe cases, it may also compromise the final occlusion efficacy and patient safety.

Moreover, in patients with concomitant structural abnormalities of the interatrial septum (such as atrial septal aneurysm or fibrosis), conventional techniques are more prone to induce serious complications, including atrial wall perforation or aortic perforation (7). The novel transseptal puncture guidewire (Accusafe™), provided by Synapse Medical, when used in conjunction with the steerable sheath (Navigo™), enables precise positioning of the optimal puncture site through sheath manipulation and utilizes the superior puncture performance of the guidewire to minimize needle displacement (8). This study aims to evaluate the differences in puncture accuracy and procedural efficiency between this novel Accusafe™ transseptal puncture guidewire and conventional methods through a small-scale consecutive case series, thereby providing empirical evidence for clinical practice.

## 2 Methods

### 2.1 Patient characteristics

This study consecutively enrolled 20 non-valvular atrial fibrillation (AF) patients scheduled for a one-stop procedure (AF ablation combined with LAAO). Among them, 10 patients were assigned to the conventional TSP group and 10 to the novel TSP guidewire group. All patients had no prior history of TSP. All procedures were performed under local anesthesia with intracardiac echocardiography (ICE) guidance. All 20 procedures in this study were performed by a single senior operator with over 120 cases of experience in single-stage atrial fibrillation ablation combined with left atrial appendage occlusion. Prior to this study, the operator had cumulatively completed more than 300 transseptal puncture procedures, demonstrating proficiency in both conventional techniques and novel device protocols. Five pre-trial procedures using the novel technology were conducted before patient enrollment, confirming no significant learning curve interference with study outcomes. This study was approved by the Medical Ethics Committee of The Sixth Affiliated Hospital of Kunming Medical University (Approval No.: 2024kmmykdx6f131), and written informed consent was obtained from all enrolled patients.

### 2.2 Transseptal puncture technique

All procedures were performed under local anesthesia with real-time guidance using ICE combined with fluoroscopy. Preoperative evaluation routinely included transthoracic echocardiography (TTE) and cardiac computed tomography angiography (CCTA). Multiplanar reconstruction was utilized to precisely measure the morphology, depth, and ostial diameter of the LAA. This imaging analysis facilitated planning of the optimal puncture site to establish an ideal coaxial trajectory directly to the LAA and to rule out the presence of thrombus within the LAA. In the control group of this study, conventional transseptal puncture was performed using standard puncture needles in strict accordance with routine clinical procedures for the one-stop left atrial appendage occlusion procedure. In the experimental group, transseptal puncture was conducted with the novel Accusafe™ transseptal puncture guidewire combined with the steerable Navigo™ sheath. A before-and-after comparative analysis of the two puncture protocols was carried out to compare the clinical performance of distinct instrument combinations for transseptal puncture.

#### 2.2.1 Conventional TSP procedural workflow

Routine right femoral venous access was obtained. Unfractionated heparin was administered for anticoagulation, maintaining an activated clotting time (ACT) > 250 s. An ICE catheter was advanced into the right atrium and manipulated to acquire a reference view that simultaneously clearly visualized the Superior Vena Cava (SVC), Fossa Ovalis (FO), and Left Atrium (LA), typically achieved by forming a “P"-shaped curve. A stiff guidewire was advanced into the SVC. Over this wire, the SL1 fixed-curve sheath (Swartz™, Abbott, USA), dilator, and puncture needle assembly were advanced into the SVC. Under guidance in the reference ICE view, the sheath's sidearm indicator was rotated to the 4–5 o'clock position. The entire assembly was then withdrawn synchronously. During withdrawal, the sheath orientation was kept stable, while minor ICE catheter adjustments were performed using fanning maneuvers to ensure continuous visualization of the dilator tip. When the sheath assembly reached the target position over the fossa ovalis, multiplanar ICE assessment was used to evaluate the puncture site location. Fine adjustments were made using clockwise or counter-clockwise rotation as needed until the ideal position was achieved, characterized by a stable “tent sign”. The needle was then advanced to puncture the septum. Following puncture, blood was aspirated and contrast was injected to confirm entry into the LA. Subsequently, the puncture needle was withdrawn. A guidewire was advanced into the left superior pulmonary vein, and the sheath was then advanced over the wire into the LA.

#### 2.2.2 Novel guidewire-assisted TSP procedural workflow

Routine right femoral venous access was established, followed by systemic heparinization to maintain an activated clotting time (ACT) > 250 s. After acquiring the reference ICE view using the standard technique, a stiff guidewire was advanced into the superior vena cava (SVC). The steerable sheath (Navigo™, Synaptic Medical, China) with its dilator was then advanced over the guidewire into the SVC. Under continuous guidance in the reference ICE view, the sheath’s sidearm indicator was rotated to the 4–5 o'clock position, and the steerable sheath-dilator assembly was withdrawn synchronously. Upon reaching the target position over the fossa ovalis (FO), multiplanar ICE assessment was performed. Fine adjustments were made using rotation or deflection adjustments of the steerable sheath until a stable “tent sign” was achieved. The stiff guidewire was then withdrawn. Subsequently, the transseptal puncture guidewire (Accusafe™) was advanced through the dilator to perform the puncture. Entry into the left atrium (LA) was confirmed by observing the trajectory of the guidewire’s enhanced-visibility segment under ICE or fluoroscopic guidance. Following confirmation, the guidewire was advanced into the left superior pulmonary vein. The steerable sheath was then advanced over the guidewire into the LA.

#### 2.2.3 Device implantation

Following successful TSP, LAAO was performed according to standard protocols in all patients. The types of occluder devices were evenly distributed between the two groups: the conventional TSP group and the novel transseptal puncture guidewire TSP group each implanted 7 disc-occluders and 3 plug-occluders.

#### 2.2.4 Study endpoints and definitions

**Figure 1:**
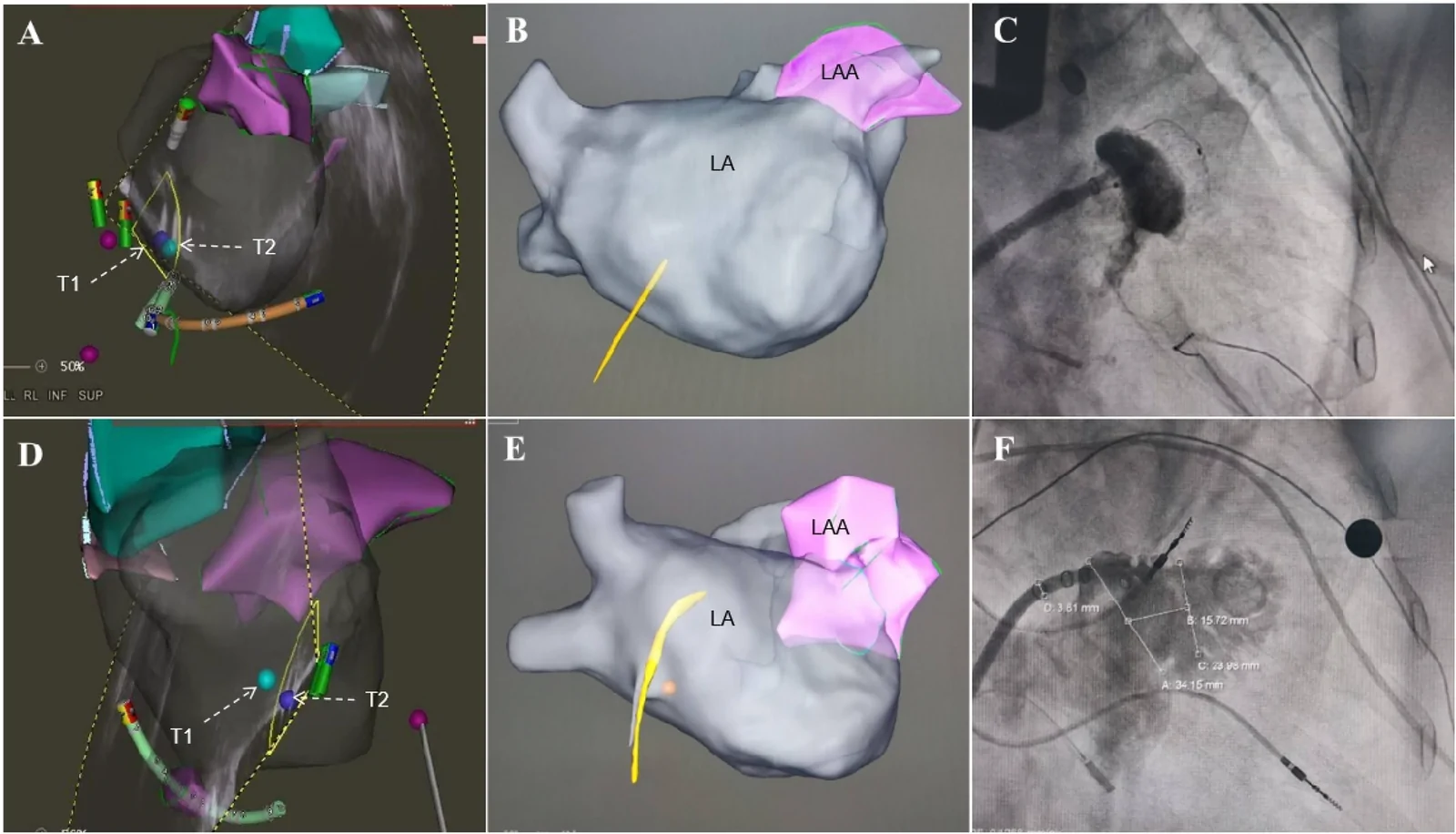
CARTO 3D reconstruction and ICE guidance during LAAO. **(A,D)** Target puncture site (T1, dark blue) versus actual puncture site (T2, light blue) in Novel Guidewire-Assisted TSP **(A)** and Conventional TSP **(D) (B,E)** Three-dimensional anatomical models depicting the Novel Guidewire-Assisted TSP **(B)** and Conventional Needle TSP **(E)** approaches: gray = left atrium, yellow = transseptal trajectory, pink = LAA. **(C,F)** Angiographic coaxiality assessment: Optimal sheath-LAA alignment (angle <30°) with Novel Guidewire-Assisted TSP **(C)** achieving successful device implantation, versus suboptimal coaxiality (angle >30°) in Conventional TSP **(F)** resulting in failed device placement.

First-pass success rate: Defined as successful puncture achieved on the initial attempt without requiring repeat puncture or equipment repositioning. Transseptal puncture time: The procedural duration from initiation of sheath withdrawal from the SVC until complete advancement of the sheath into the LA. Sheath displacement: Quantitatively measured under ICE guidance using the CARTO 3D mapping system. The peak tenting position of the needle/sheath tip on the interatrial septum at puncture initiation (point T1 in Figures 1A,D) was marked and compared to the final sheath tip position post-successful puncture (point T2 in Figures 1A,D). The linear distance (mm) between T1 and T2 was calculated. Coaxiality assessment: Following sheath placement within the LAA, angiography was performed to measure the angle between the central axis of the delivery sheath and the central axis of the LAA. An angle <30° was considered satisfactory coaxiality. LAAO time: Defined as the interval from delivery sheath advancement into the LA until confirmation of satisfactory device position by angiography and/or ICE followed by device release.

To standardize the endpoint definitions for LAAO trials, occlusion efficacy was defined using a five-tier sealing grading system based on the degree of residual peri-device leak and device stability (9), as follows: Seal Grade I (optimal seal): complete LAA occlusion with no residual peri-device leak; Seal Grade II (adequate seal): presence of a minimal peri-device residual leak with a jet diameter ≤1 mm; Seal Grade III (suboptimal seal): residual leak with a jet diameter >1 mm but ≤3 mm; Seal Grade IV (inadequate seal): significant residual leak with a jet diameter >3 mm; and Seal Grade V (device failure): failure of successful device implantation, including device malposition, embolization, or inability to deploy.

### 2.3 Statistical analysis

Statistical analyses were performed using SPSS software (version 22.0). Continuous data are expressed as mean ± standard deviation ($\bar{x}$ ± s). Intergroup comparisons utilized the independent samples t-test and the non-parametric Mann–Whitney U test (Wilcoxon rank-sum test). Given the small sample size (*n* = 10 per group), the first-pass success rate was compared between groups using a two-sided Fisher’s exact test, with all analyses treated as exploratory. A *P* < 0.05 was considered statistically significant.

## 3 Results

### 3.1 Comparison of baseline characteristics

The baseline characteristics of both patient groups are presented in Table 1. The groups were comparable in age (conventional group: 71 ± 9.14 years vs. novel group: 69.8 ± 5.41 years) and predominantly male (70% each). Regarding stroke and bleeding risks, the conventional group showed numerical differences in CHA₂DS₂-VASc score (3.8 ± 1.03 vs. 4.3 ± 0.82) and HAS-BLED score (2.6 ± 1.07 vs. 3.1 ± 0.88) compared to the novel group, but exhibited comparable overall baseline risk profiles. No significant intergroup difference was observed in left atrial anteroposterior diameter (conventional group: 40.1 ± 4.86 mm vs. novel group: 43.2 ± 5.40 mm).

**Table 1:** Comparison of baseline and clinical characteristics between patient groups.

Baseline clinical characteristics	Conventional TSP group (*n* = 10)	Transseptal guidewire TSP group (*n* = 10)
Age (mean ± SD, years)	71.0 ± 9.14	69.8 ± 5.41
Male, *n* (%)	7 (70%)	7 (70%)
CHA2DS2-VASc score (mean ± SD)	3.8 ± 1.03	4.3 ± 0.82
HAS-BLED score (mean ± SD)	2.6 ± 1.07	3.1 ± 0.88
Left atrial anteroposterior diameter (mm)	40.1 ± 4.86	43.2 ± 5.40
Atrial fibrillation type, *n* (%)		
Paroxysmal	1 (10%)	1 (10%)
Persistent	7 (70%)	9 (90%)
permanent	2 (20%)	0 (0%)
Interatrial septal characteristics, *n* (%)		
Normal	10 (100%)	8 (80%)
Atrial septal aneurysm	0 (0%)	1 (10%)
Fibrosis/thickening	0 (0%)	1 (10%)
LAA anatomy, *n* (%)		
Chicken wing	7 (70%)	7 (70%)
Non-angulated (windsock/cactus/cauliflower)	3 (30%)	3 (30%)

In AF type distribution, the novel group consisted predominantly of persistent AF (90%), while the conventional group included some permanent AF cases (20%). Regarding interatrial septal anatomy, all conventional group patients had normal septa, whereas the novel group contained one case of atrial septal aneurysm and one case of fibrosis/thickening. For LAA morphology, both groups were dominated by Chicken Wing morphology (70% each), with the remaining cases classified as non-Chicken Wing types (Windsock/Cactus/Cauliflower).

### 3.2 Puncture success rate and safety

All patients successfully underwent TSP without major procedure-related complications. In the novel TSP guidewire group, 100% of punctures (10/10) were achieved on the first attempt, eliminating risks associated with repeated punctures or positional adjustments. In contrast, the conventional TSP group demonstrated a lower first-pass success rate of 70% (7/10), frequently requiring multiple minor sheath adjustments. This numerical advantage in the novel group did not reach statistical significance by Fisher's exact test (two-sided *P* = 0.105); nevertheless, the results suggest a potential trend favoring the novel guidewire. All comparisons were exploratory given the small sample size (*n* = 10 per group), and larger studies are warranted to confirm this observation (Table 2).

**Table 2:** Procedural efficacy of conventional versus novel transseptal puncture guidewire techniques.

Procedural and perioperative outcomes	Conventional TSP group (*n* = 10) (*n* = 10)	Transseptal guidewire TSP group (*n* = 10)
First-pass succes, *n* (%)	7 (70.0%)	10 (100.0%)
Sheath displacement, mm (mean ± SD)	2.8 ± 0.92	0.5 ± 0.71
Alignment (<30°), *n* (%)	7 (70.0%)	10 (100.0%)
TSP time, seconds (mean ± SD)	224.8 ± 47.31	196.5 ± 15.33
Radiation exposure time, seconds (mean ± SD)	31.2 ± 11.53	27.2 ± 8.08
Radiation dose (mGy, mean ± SD)	16.2 ± 5.96	15.4 ± 4.79
LAAO time, minutes (mean ± SD)	37.90 ± 6.98	31.41 ± 6.50
Seal grade I (optimal seal), *n* (%)	5（50.0%）	8（80.0%）
Seal grade II (adequate seal), *n* (%)	4（40.0%）	1（10.0%）
Seal grade III (suboptimal seal), *n* (%)	0（0.0%）	0（0.0%）
Seal grade IV (inadequate seal), *n* (%)	0（0.0%）	0（0.0%）
Seal grade V (device failure), *n* (%)	1（10.0%）	1（10.0%）
Complications, *n* (%)	0（0.0%）	0（0.0%）

### 3.3 Sheath displacement and coaxial alignment

Sheath displacement, a critical determinant of puncture accuracy, was significantly greater in the conventional TSP group (2.8 ± 0.92 mm) compared to minimal displacement observed with the novel transseptal approach (0.5 ± 0.71 mm; *P* < 0.05) (Table 2). This marked reduction in displacement with the novel guidewire technique enabled optimal long-axis coaxial alignment between the delivery sheath and LAA (Figure 1), achieving superior coaxiality (100% vs. 70%, *P* < 0.05) that not only substantially reduced delivery system resistance for smoother device advancement and deployment but crucially ensured device expansion at the ideal axial position, thereby maximizing anchor stability and endothelial coverage while fundamentally mitigating peri-device leakage (PDL) risk.

### 3.4 Procedural efficiency

Transseptal puncture time served as a key metric for procedural efficiency. The novel guidewire-assisted TSP group demonstrated a mean puncture time of 196.5 ± 15.33 seconds, representing an approximately 14.4% reduction compared to the conventional TSP group (224.8 ± 47.31 s) (Table 2). Notably, the novel technique not only expedited the procedure but also exhibited a smaller SD, indicating enhanced reproducibility and operator consistency. This improvement stems from its integrated design, which reduces intraprocedural instrument exchanges and repositioning steps, thereby optimizing workflow and minimizing operator/patient exposure time.

Further analysis revealed that this puncture precision significantly shortened overall LAAO duration. The novel guidewire group achieved a mean LAAO time of 31.41 ± 6.50 min, significantly lower than the conventional group’s 37.90 ± 6.98 min (*P* < 0.05). Although radiation dose showed no significant intergroup difference (15.40 ± 4.79 mGy vs. 16.20 ± 5.96 mGy, *P* > 0.05) and remained within acceptable ranges for both cohorts, the novel technique's reduction in total procedural time effectively mitigated patient intraoperative risks (Table 2).

### 3.5 Occlusion efficacy

Regarding occlusion efficacy, ideal seal (Grade I) was achieved in 8/10 (80%) patients in the novel guidewire-assisted TSP group and 5/10 (50%) in the conventional TSP group, with an additional 4/10 (40%) in the conventional group achieving adequate seal (Grade II). Notably, one device failure (Grade V) occurred in each group. In the novel group, failure resulted from an LAA orifice exceeding the device's anatomical compatibility, an independent constraint unrelated to puncture technique. In the conventional group, failure was caused by sheath displacement with insufficient coaxiality during delivery. Overall, 18 of 20 patients (90%) achieved favorable occlusion outcomes (Grades I–II), while the remaining 2 cases represented device failures due to distinct, unrelated mechanisms (Table 2). The novel transseptal puncture guidewire technique demonstrated enhanced procedural stability, reduced operative time, and improved safety, warranting further investigation.

## 4 Discussion

This study enrolled 20 patients for a preliminary investigation of the clinical utility of a novel transseptal puncture guidewire combined with a steerable sheath in LAAO. Results demonstrated that this technique significantly enhanced procedural stability and efficiency compared to conventional methods. Conventional TSP techniques exhibit inherent limitations due to their heavy reliance on operator experience. First, manual manipulation inherently restricts precision, hindering accurate localization of subtle anatomical structures like the fossa ovalis while rendering the procedure susceptible to interference from respiration, cardiac motion, and vascular pulsation (7). Second, the lack of effective support often necessitates multiple blind adjustments during conventional TSP, prolonging procedural duration and increasing the risk of atrial septal injury (10). In this study, the conventional group’s 70% first-pass success rate and significant sheath displacement (2.8 ± 0.92 mm) directly reflect these limitations.

In contrast, the novel transseptal puncture guidewire technique provides rigid support throughout the puncture trajectory via its integrated design. Data confirmed that this approach strictly limited sheath displacement to 0.5 ± 0.71 mm while achieving 100% first-pass success and 100% optimal coaxial alignment (<30°). This submillimeter-level stability not only eliminated risks associated with repeat punctures (e.g., pericardial tamponade or vascular injury) (11, 12) but more pivotally established an ideal delivery pathway for subsequent occluder deployment. The optimal coaxiality ensured devices followed the natural anatomical curvature into the left atrial appendage, substantially reducing delivery resistance and minimizing risks of device embolization or cardiac perforation (13, 14).

In terms of procedural efficiency, the novel guidewire technique demonstrated significant advantages. It reduced puncture time by approximately 14.4% (196.5 ± 15.33 s vs. 224.8 ± 47.31 s), with this efficiency gain directly translating to shorter total procedural duration (31.41 ± 6.50 min vs. 37.90 ± 6.98 min).

The AccuSafe™ transseptal puncture guidewire employed in this study effectively resolves conventional TSP limitations through its innovative visualizable-tip/flexible-distal-segment configuration. This integrated wire-needle system features a sharpened penetrating tip for effective tissue traversal and a unique J-tip design that intrinsically ensures safety by mechanically constraining inadvertent injury to the left atrial appendage or pulmonary veins, while simultaneously ensuring submillimeter-range sheath displacement (0.5 ± 0.71 mm) and achieving 100% optimal coaxial alignment (<30°). When coupled with a steerable sheath, the system enables real-time curvature control for millimeter-precise puncture site selection—this novel transseptal methodology not only streamlines procedural workflow and reduces operative time but fundamentally establishes a stable delivery pathway foundation for subsequent precise occluder deployment, particularly suited for safety-critical LAAO procedures.

This study has several limitations. First, the limited sample size (*n* = 20) and single-center design may introduce selection bias. Second, non-randomized allocation could potentially compromise objective intergroup comparison of occlusion efficacy. Additionally, the simultaneous adoption of the novel steerable sheath and novel puncture guidewire means the synergistic effect of both devices collectively contributes to the observed procedural performance benefits. Current data cannot fully distinguish their independent contributions. Subsequent single-factor controlled studies under standardized sheath conditions will further validate the standalone value of the Accusafe™ guidewire.

## 5 Conclusion

In summary, this exploratory study demonstrates that the novel transseptal puncture guidewire technique is feasible and may enhance procedural efficiency during LAAO. The observed improvements in procedural stability, precision, and safety profile suggest that this approach has the potential to address some limitations associated with conventional puncture methods. However, given the small sample size and the exploratory nature of this comparison, these findings should be interpreted as preliminary. Extreme anatomical variants of the left atrial appendage remain independent determinants of final occlusion efficacy. Future larger-scale, multicenter randomized controlled trials are warranted to confirm these procedural advantages and to investigate potential long-term clinical benefits.

## Data Availability

The raw data supporting the conclusions of this article will be made available by the authors, without undue reservation.
